# An Electronic Health Record–Based Intervention to Facilitate Primary Care Referrals to the Special Supplemental Nutrition Program for Women, Infants, and Children: Retrospective Cohort Study

**DOI:** 10.2196/87608

**Published:** 2026-10-07

**Authors:** Kimberly Montez, Melissa C Kay, Alysha J Taxter, Kristina H Lewis

**Affiliations:** 1 Department of Pediatrics, Wake Forest University School of Medicine, One Medical Center BlvdWinston-Salem, NC, 27157, United States, 1 336-716-2588; 2 Department of Social Sciences & Health Policy, Wake Forest University School of Medicine, Winston-Salem, United States; 3 Department of Epidemiology & Prevention, Wake Forest University School of Medicine, Winston-Salem, United States; 4 Department of Pediatrics, Nationwide Children's Hospital, Columbus, OH, United States; 5 Department of Implementation Science, Wake Forest University School of Medicine, Winston-Salem, United States; 6 Department of Surgery, Wake Forest University School of Medicine, Winston-Salem, United States

**Keywords:** WIC, food insecurity, electronic health records, referrals, pediatrics

## Abstract

**Background:**

Despite the benefits of the Special Supplemental Nutrition Program for Women, Infants, and Children (WIC), many eligible children remain unenrolled.

**Objective:**

This study evaluated responses to an electronic health record (EHR)–embedded federal nutrition program (FNP) participation screening and WIC referral tool implemented within 8 clinics during well-child visits for children younger than 5 years of age.

**Methods:**

Structured EHR data from July 2020 to October 2023 were extracted to summarize screening, referral acceptance, WIC enrollment outcomes, and patient demographics. Multivariable logistic regression was used to examine patient-level predictors of WIC nonenrollment at the first screening, referral acceptance, and enrollment among those accepting a referral, with analyses stratified by clinic type.

**Results:**

Among 5385 children, 51.8% (n=2785) were newborn-aged, 36.6% (n=1969) were Hispanic, and 87.8% (n=4726) were Medicaid-insured. The mean age at the initial screening was 10.7 (SD 14.2) months. Among screened patients (n=3606), 34% (n=1235) were not enrolled in WIC at the first screening. Medicaid coverage, an academic clinic setting, non-Hispanic Black or Hispanic race, and ethnicity were associated with lower odds of WIC nonenrollment. Among those not enrolled with a documented response to referral (n=819), 59% (n=488) accepted the referral. Acceptance was higher among non-Hispanic Black and Hispanic patients, newborn-aged patients, those with Medicaid coverage, and those seen in academic clinics. Among referral acceptors with follow-up (n=438), 61% (n=265) were enrolled at the last screening, with higher odds among newborn-aged patients and those in academic clinics.

**Conclusions:**

An EHR-based automated intervention can facilitate screening and referral to WIC. WIC participation at the first screening, referral acceptance, and enrollment after referral varied by sociodemographic characteristics, suggesting opportunities to improve equitable access through health care system–based approaches.

## Introduction

The Special Supplemental Nutrition Program for Women, Infants, and Children (WIC) is a federal nutrition program (FNP) that provides access to nutritious foods, nutrition education, lactation support, and health care referrals for eligible low-income pregnant and lactating individuals, as well as infants and children under 5 years of age. Participation in WIC is associated with numerous health benefits, including improvements in dietary quality and reductions in the risk of prematurity, low birth weight, childhood obesity, and food insecurity [1,2], all of which disproportionately affect racial and ethnic minority populations due to longstanding structural racism [3,4]. Despite these health benefits of WIC participation, many eligible infants and children remain unenrolled.

In 2023, the most recent year in which data are available, only 56% of the eligible 11.8 million women, infants, and children participated in WIC, resulting in benefit underutilization and missed opportunities for improvements in health outcomes [5]. Since 2011, when it peaked at 63%, WIC participation has been declining [6]. Well-documented barriers to participation in WIC include lack of knowledge about WIC or eligibility status, lack of time or transportation to attend in-person visits, cultural and language barriers, negative experiences with WIC clinics, challenges in redeeming benefits, and stigma, among other barriers [7-9]. WIC program flexibilities implemented during the COVID-19 pandemic, such as remote benefit issuance, eased some of the administrative burden of participation and resulted in modest increases in participation [10]. To further expand outreach, increase enrollment in WIC, and ultimately improve health outcomes, cross-sector strategies are necessary [11].

Primary care clinics are uniquely positioned to counsel patients about WIC, facilitate WIC referrals, and encourage enrollment during standard discussions about nutrition at well visits. Providing referrals to WIC in primary care settings represents an evidence-based intervention for reducing food insecurity [12-14]. Several national organizations, including the American Academy of Pediatrics and the Food Research & Action Center, recommend that pediatricians refer patients to WIC [15-17]. Several studies have evaluated care coordination between the health care system and WIC to improve outcomes, such as responsive parenting, and have found that, while few have impacted weight outcomes, integrated services were acceptable among participants [18-21]. However, few have implemented and evaluated WIC participation screening and direct referrals to WIC from the health care system [22-24]. This study evaluated responses to an electronic health record (EHR)–embedded FNP participation screening and WIC referral tool, including screening results, referral acceptance, and WIC enrollment, and examined patient-level correlates and differences by clinic type.

## Methods

### Study Design and Setting

This retrospective cohort study was part of a mixed methods investigation in which our team expanded an innovative EHR-based FNP participation screening and referral tool. The tool assessed both WIC and the Supplemental Nutrition Assistance Program (SNAP) enrollment and provided WIC referrals at 8 primary care clinics, including 1 academic pediatric clinic, 5 private pediatric clinics, and 2 family medicine clinics, between July 2020 and October 2023. The study took place within Atrium Health Wake Forest Baptist, a large health care system serving a racially and ethnically diverse patient population in the western portion of North Carolina, which uses a fully integrated EHR system (Epic). See McCall et al [24] for a detailed description of the patient demographics of each of these health care clinics. The Wake Forest University School of Medicine Institutional Review Board approved this study.

### Electronic Screening and Referrals

Beginning in July 2020, an EHR-embedded tool was implemented during well-child visits at the point of care in one academic clinic, which was a quality improvement project that tested various Plan-Do-Study-Act cycles [22]. Given the success of this project, the tool was expanded in 2021 to 7 other nonacademic clinics, which is the focus of the current study. For the 7 nonacademic clinics, the attending physicians and advanced practice providers exclusively provided care, with patient rooming and screening generally performed by medical assistants (MAs) and other support staff. Within these 7 clinics, considering rooming workflows and the preferences of providers, who did not prefer to have screening embedded in the note template or to perform screening themselves, an automated alert prompted MAs to screen caregivers for FNP participation during the rooming process and to assess family interest in a referral to WIC if not already participating. Given that Medicaid eligibility conferred WIC eligibility, the alert was programmed only to appear for visits with children below 5 years of age who had Medicaid or were uninsured or lived in Forsyth County, North Carolina, the location of our WIC partner. In addition, the alert appeared for those who had not been screened in the prior 6 months and thus did not appear at every visit. The FNP screening question asked: “Does your child currently participate in the WIC or SNAP programs? SNAP is also known as Food Stamps.” Self-reported participation response options of FNP were as follows: WIC only, SNAP only, both WIC/SNAP, neither WIC/SNAP, unsure, or did not ask. For participants who answered the initial FNP screening with the response options of “SNAP only,” “neither,” or “unsure,” an additional automated alert prompted the MA to assess caregiver interest in WIC referral with the following question: “WIC is a free program through the health department that provides food and nutrition support for children from birth through 4 years old. Can we give your information to WIC so they can contact you about enrolling?” Response options were: “Yes, family approves referral for WIC contact” or “No, family does not approve referral for WIC contact.” Prior to activation of the new screening tool within the EHR, research staff visited all clinics in person and conducted hands-on training sessions with clinic staff on how to effectively screen and refer using the tool, as well as how to counsel families. A laminated 1-page job aid was also provided with multiple copies at nursing workstations in each clinic. The job aid included contact information for research team members so that clinic staff or clinic managers could reach out with questions or concerns. Additionally, after the tool was live in the EHR, research staff members followed up regularly through email for 6 months with clinical practice managers to actively ask about any questions or difficulties that staff had encountered with screening.

At the single academic pediatric clinic in our sample, most of the clinical care is provided by residents and advanced practice providers, and the clinical workflows do not typically involve MAs performing screening duties. At this clinic, WIC screening was operationalized differently to increase screening and referral uptake, with providers (physicians, usually residents) being prompted to screen for FNP participation via an EHR-embedded tool within the well-child visit note template, which has been previously described [22,24]. At the academic clinic, FNP screening was prompted at every well-child visit for children under 5 years because the clinic serves almost exclusively FNP-eligible patients. The same training for the nonacademic clinic was also provided for the academic clinic. In addition, at the start of every new resident block (eg, every 4 wk), providers were given an orientation at morning report, which included how to effectively screen and refer to WIC using the EHR.

For consenting families at all 8 clinics, automated referrals were sent directly to WIC staff via an EHR in-basket message to begin enrollment, as previously described [6,23]. For the study period, structured data were extracted from the pediatric patient’s EHR for screening and referral results as well as demographics, including age, race, ethnicity, insurance type, preferred language for health care use, sex, and clinic in which screening occurred. Race, ethnicity, and preferred language for health care use were extracted from self-reported fields within the EHR. Due to missing demographic data, 112 records were excluded for the first screening instance and 94 for the last screening instance.

### Study Demographics

Extracted demographics, as noted above, included child age and sex because WIC participation rates vary by age [6], and male sex has been associated with longer WIC participation duration [25]. For age, we categorized pediatric patients as being newborn-aged (≤3 months old) or not (>3 months old), given that the age distribution of the sample was highly concentrated in early infancy and pediatric visit frequency is particularly high during the newborn phase. Race and ethnicity were included because the percentage of the eligible population participating in WIC varies by race and ethnicity, with non-Hispanic Black and Hispanic WIC-eligible individuals more likely to participate in WIC than non-Hispanic White individuals [6]. For analytic purposes, the race and ethnicity categories were Hispanic, non-Hispanic Black, and non-Hispanic White. We included other races and ethnicities in a combined category that included Asian, American Indian/Alaska Native, and Native Hawaiian/Pacific Islander, but we did not interpret them in our results, given small clinic populations of each of these races and ethnicities. Insurance type was categorized as Medicaid or non-Medicaid; non–Medicaid insured patients included those who were uninsured or had commercial or other third-party insurance types. The language preference of the caregiver for health care use, categorized as English, Spanish, or “other,” was included because it may be associated with barriers to participation [9,26]. Given the small number of pediatric patients with a primary language other than Spanish or English, we did not interpret the results of the “other” category. Clinic sites were grouped according to the method by which screening took place, such that the 7 clinics using MAs to screen via the automated alert were considered as one category, while one pediatric academic clinic using providers to screen via the note template was considered as a separate category.

### Outcomes and Data Analysis

The primary outcomes were (1) likelihood of WIC nonenrollment at the first screening, (2) likelihood of accepting a referral, and (3) likelihood of enrolling in WIC among those accepting a referral. We characterized the initial FNP screening and WIC referral status using the response categories listed above. Among participants for whom the FNP screening was prompted at more than one visit during the study period, we also characterized those who transitioned from reporting no WIC participation (eg, initial screening results of SNAP only, neither WIC/SNAP) to subsequently reporting WIC participation at the final screen as becoming enrolled in WIC. Because follow-up intervals between well-child visits vary, and to allow sufficient time for WIC referrals to potentially result in enrollment, outcomes were defined using the last documented screening encounter during the study period rather than a fixed follow-up window.

Descriptive statistics were used to summarize demographic characteristics and screening and referral responses. Because this study aimed to evaluate and optimize a novel clinical screening and referral process, we used multivariable logistic regression to assess associations with screening and referral outcomes. All models adjusted for child age, sex, race and ethnicity, preferred language, insurance type, year of screening, and clinic type. In analyses of subsequent WIC enrollment at the last screening encounter, we additionally adjusted for days between the first and last screening visits as an adjustment covariate to account for variation in follow-up time and opportunity for enrollment after referral. Because the primary outcome defined WIC enrollment as a receipt of WIC alone or in combination with SNAP, SNAP participation could not be modeled as a separate, mutually exclusive covariate. Models additionally adjusted for the year of the screening encounter to account for temporal trends during the study period. Because the academic clinic implemented screening earlier than the nonacademic clinics and some individual years contained sparse cells for certain outcomes, screening year was categorized into 2 periods (2020‐2021 and 2022‐2023) for regression analyses. We first fit models including clinic type as an independent variable and then conducted stratified analyses by fitting the models separately among patients from academic and nonacademic clinics. In stratified models with sparse cells, when a categorical predictor level had zero outcome events (complete separation), that level was omitted from the model, and comparisons were estimated among remaining levels with observed events. Results are reported as odds ratios (ORs) with 95% CIs. Robust SEs were used to account for potential heteroskedasticity. The results were considered statistically significant at a 2-tailed *P*<.05. Data cleaning and analysis were completed using SAS version 9.4 (SAS Institute, Inc) and Stata version 18.5 (StataCorp).

### Ethical Considerations

The Wake Forest University School of Medicine Institutional Review Board approved this study (IRB00078127). As this was a retrospective cohort, EHR-based study, participants’ informed consent was waived, and compensation was not provided.

## Results

There were 5497 unique records in which the FNP screening prompt appeared at ≥1 visit. Of these, 5385 (98%) records had complete data for all demographic measures of interest and were included in analyses. The mean age of the pediatric patient at the initial screening was 10.7 (SD 14.2; range: 0.03‐59.2) months, and 51.8% (n=2785) were newborn-aged. Just over half of patients were male (n=2780, 51.6%), 36.6% (n=1969) were Hispanic, 76.4% (n=4113) preferred English for health care use, and 87.8% (n=4726) had Medicaid insurance (Table 1). Few (n=129, 2.4%) reported participation in SNAP only, while 44% (n=2369) were already enrolled in WIC or both WIC and SNAP. Because WIC screening was embedded within the provider note template in the academic clinic, we were unable to determine when screening was prompted but not completed in that setting; therefore, screening completion could only be reliably quantified for the nonacademic clinics, where an automated alert allowed explicit documentation of completed or dismissed screening. Within the nonacademic clinics, 1108 (38%) records completed the screening for WIC participation. Among all patients who were screened (n=3606), 66% (n=2371) reported participation in WIC or both WIC and SNAP at the first screening encounter, while 34% (n=1235) reported no participation in FNP or were unsure. Figure 1 details sample sizes at each step.

**Table 1.** Patient characteristics by Special Supplemental Nutrition Program for Women, Infants, and Children (WIC) enrollment status at the first screening encounter, 2020‐2023 (N=5385).

Demographic characteristic	All patients (N=5385), n (%)	Academic clinic (n=2498)		Nonacademic clinics (n=2887)		
		WIC/WIC and SNAP^a^ (n=1857), n (%)	Not participating in WIC (n=641), n (%)	WIC/WIC and SNAP (n=514), n (%)	Not participating in WIC (n=594), n (%)	Not screened (n=1779), n (%)
Sex						
Female	2605 (48.4)	886 (47.7)	313 (48.8)	252 (49.0)	306 (51.5)	848 (47.7)
Male	2780 (51.6)	971 (52.3)	328 (51.2)	262 (51.0)	288 (48.5)	931 (52.3)
Race and ethnicity						
Non-Hispanic White	1171 (21.8)	149 (8.0)	73 (11.4)	115 (22.4)	256 (43.1)	578 (32.5)
Non-Hispanic Black	1788 (33.2)	554 (29.8)	119 (18.6)	254 (49.4)	190 (32.0)	671 (37.7)
Hispanic	1969 (36.6)	1052 (56.7)	377 (58.8)	101 (19.7)	89 (15.0)	350 (19.7)
Non-Hispanic Other^b^	457 (8.5)	102 (5.5)	72 (11.2)	44 (8.6)	59 (9.9)	180 (10.1)
Newborn (<3 mo)						
Yes	2785 (51.8)	1306 (70.3)	426 (66.5)	194 (37.7)	250 (42.0)	609 (34.2)
No	2600 (48.3)	551 (29.7)	215 (33.5)	320 (62.3)	344 (57.9)	1170 (65.8)
Preferred language						
English	4113 (76.4)	1094 (58.9)	351 (54.8)	467 (90.9)	564 (95.0)	1637 (92.0)
Spanish	1208 (22.4)	738 (39.7)	269 (42.0)	45 (8.8)	28 (4.7)	128 (7.2)
Other	64 (1.2)	25 (1.4)	21 (3.3)	2 (0.4)	2 (0.3)	14 (0.8)
Insurance						
Medicaid	4726 (87.8)	1814 (97.7)	573 (89.4)	494 (96.1)	433 (72.9)	1412 (79.4)
Non-Medicaid	659 (12.2)	43 (2.3)	68 (10.6)	20 (3.9)	161 (27.1)	367 (20.6)
Year at the first screening						
2020‐2021	2497 (46.4)	1140 (61.4)	366 (57.1)	326 (63.4)	222 (37.4)	443 (24.9)
2022‐2023	2888 (53.6)	717 (38.6)	275 (42.9)	188 (36.6)	372 (62.6)	1336 (75.1)
SNAP-only participation^c^						
Yes	129 (2.4)	—^d^	68 (10.6)	—	61 (10.3)	—
No	5256 (97.6)	—	573 (89.4)	—	533 (89.7)	—

a SNAP: Supplemental Nutrition Assistance Program.

b Other race and ethnicity categories included Asian, American Indian/Alaska Native, and Native Hawaiian/Pacific Islander.

c SNAP-only participation is shown. Participants receiving both WIC and SNAP are included in the WIC participation category used for study outcomes and are therefore not represented in this row.

d Not applicable.

**Figure 1.**
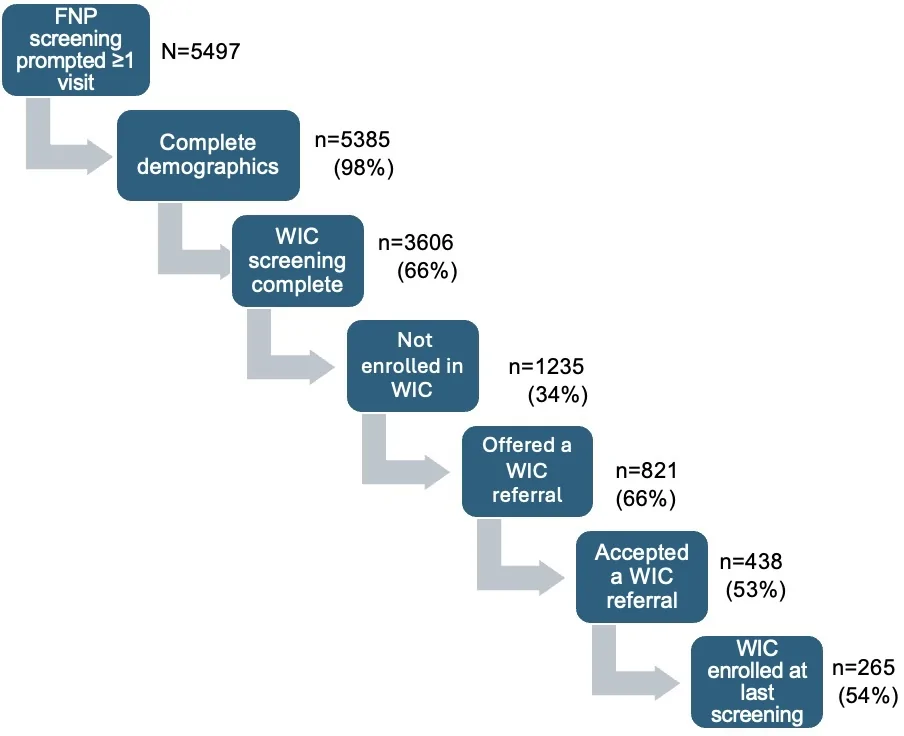
Flow of the analytic sample across Special Supplemental Nutrition Program for Women, Infants, and Children (WIC) screening, referral, and enrollment outcomes. WIC enrolled at the last screening at the time of data extraction. FNP: federal nutrition program.

Table 2 presents factors associated with WIC nonenrollment at the first screening encounter; ORs less than 1 indicate lower odds of nonenrollment (ie, greater likelihood of being enrolled in WIC). In the overall model, Medicaid coverage was strongly associated with lower odds of nonenrollment compared with non-Medicaid insurance, and patients attending academic clinics had lower odds of nonenrollment than those seen at nonacademic clinics. Compared with non-Hispanic White patients, non-Hispanic Black and Hispanic patients were also less likely to be unenrolled. Newborn-aged patients had modestly lower odds of nonenrollment relative to older children. In contrast, the odds of WIC nonenrollment increased over time, with higher odds observed in 2022 to 2023 compared with 2020 to 2021. Sex and preferred language were not significantly associated with nonenrollment. Stratified analyses by clinic type demonstrated generally similar patterns, with some attenuation of associations in subgroup models.

**Table 2.** Multivariable logistic regression of the odds of Special Supplemental Nutrition Program for Women, Infants, and Children (WIC) nonenrollment at the first screening encounter, overall and stratified by clinic type (N=3606)^a^.

Characteristic	Overall (N=3606), OR^b^ (95% CI)	Academic clinic (n=2498), OR (95% CI)	Nonacademic clinics (n=1108), OR (95% CI)
Sex (Ref: female)			
Male	0.96 (0.83-1.11)	0.99 (0.82-1.19)	0.89 (0.69-1.16)
Race and ethnicity (Ref: Non-Hispanic White)			
Non-Hispanic Black	0.45 (0.36-0.78)^c^	0.50 (0.35-0.70)^c^	0.43 (0.31-0.59)^c^
Hispanic	0.75 (0.59-0.96)^c^	0.88 (0.63-1.21)	0.64 (0.41-1.00)^c^
Newborn (Ref: no)			
Yes	0.81 (0.69-0.96)^c^	0.83 (0.67-1.03)	0.78 (0.59-1.03)
Preferred language (Ref: English)			
Spanish	0.95 (0.77-1.19)	1.01 (0.80-1.28)	0.60 (0.33-1.10)
Insurance (Ref: Non-Medicaid)			
Medicaid	0.18 (0.13-0.24)^c^	0.21 (0.14-0.32)^c^	0.14 (0.08-0.24)^c^
Year at the first screening (Ref: 2020‐2021)			
2022‐2023	1.63 (1.40-1.91)^c^	1.26 (1.03-1.53)^c^	2.65 (2.04-3.46)^c^
Academic clinic (Ref: no)			
Yes	0.37 (0.31-0.45)^c^	—^d^	—

a Categories for other race and ethnicity and other language were suppressed due to small sample sizes. Models were adjusted for screening year to account for temporal trends during the study period (2020‐2023).

b OR: odds ratio.

c These estimates indicate statistical significance at *P*<.05.

d Not applicable.

Among participants who were not enrolled in WIC at the first screening encounter (n=1235), 66% (n=815) were offered a referral, and 819 had a documented response regarding referral acceptance. Table 3 presents factors associated with accepting a WIC referral among these patients. In the overall model, non-Hispanic Black and Hispanic patients had higher odds of accepting a referral compared with non-Hispanic White patients. Newborn-aged patients were more likely to accept a referral than older children. Medicaid coverage was associated with substantially higher odds of referral acceptance compared with non-Medicaid insurance. In contrast to patterns observed for nonenrollment, patients seen in academic clinics had higher odds of accepting a referral than those seen in nonacademic clinics. Sex, preferred language, and screening year were not significantly associated with referral acceptance overall. Stratified analyses by clinic type showed generally similar patterns, although CIs widened in subgroup models.

**Table 3.** Multivariable logistic regression of the odds of accepting a Special Supplemental Nutrition Program for Women, Infants, and Children (WIC) referral among screened patients with a documented response (N=819)^a^.

Patient characteristics	Overall (N=819), OR^b^ (95% CI)	Academic clinic (n=503), OR (95% CI)	Nonacademic clinics (n=316), OR (95% CI)
Sex (Ref: female)			
Male	0.76 (0.53-1.08)	0.84 (0.52-1.33)	0.71 (0.41-1.22)
Race and ethnicity (Ref: non-Hispanic White)			
Non-Hispanic Black	2.85 (1.71-4.75)^c^	3.04 (1.37-6.75)^c^	2.70 (1.35-5.35)^c^
Hispanic	2.06 (1.19-3.58)^c^	2.15 (1.03-4.47)^c^	2.09 (0.83-5.25)
Newborn (Ref: no)			
Yes	2.76 (1.89-4.02)^c^	3.14 (1.93-5.11)^c^	2.68 (1.43-5.02)^c^
Preferred language (Ref: English)			
Spanish	1.74 (1.02-2.99)^c^	1.63 (0.91-2.91)	2.79 (0.78-10.00)
Insurance (Ref: non-Medicaid)			
Medicaid	4.51 (2.74-7.44)^c^	3.07 (1.48-6.40)^c^	6.22 (2.60-14.91)^c^
Year at the first screening (Ref: 2020‐2021)			
2022‐2023	1.18 (0.83-1.68)	1.51 (0.93-2.44)	0.91 (0.52-1.57)
Academic clinic (Ref: no)			
Yes	4.78 (3.23-7.06)^c^	—^d^	—

a Sample restricted to patients who were screened for WIC participation and had a documented response regarding referral acceptance. Models were adjusted for child age, sex, race and ethnicity, preferred language, insurance type, year of first screening, and clinic type.

b OR: odds ratio.

c These estimates indicate statistical significance at *P*<.05.

d Not applicable.

Among those who accepted a WIC referral and had a documented follow-up response (n=438), 61% (n=265) were enrolled in WIC at the last screening encounter. Table 4 presents factors associated with subsequent WIC enrollment among these participants. In the overall model, newborn-aged patients were more likely to be enrolled in WIC at the last screening compared with older children (OR 6.80, 95% CI 3.89-11.86), and patients seen in academic clinics had higher odds of enrollment than those seen in nonacademic clinics (OR 13.29, 95% CI 4.77-37.02). In clinic-specific analyses, associations were generally consistent in the academic clinics, where newborn age remained associated with enrollment. Estimates for the nonacademic clinics were imprecise because of the smaller sample size and sparse cells; however, Medicaid insurance was associated with lower odds of enrollment compared with non-Medicaid coverage in this subgroup. Race and ethnicity estimates in the nonacademic clinics should be interpreted cautiously, as the non-Hispanic White category was omitted due to zero observed enrollment events, precluding direct comparisons with other groups. Sex, race and ethnicity, preferred language, screening year, and days between the first and last screening visits were not significantly associated with WIC enrollment overall. Due to sparse data in the nonacademic clinics, results should be interpreted with caution.

**Table 4.** Multivariable logistic regression of the odds of Special Supplemental Nutrition Program for Women, Infants, and Children (WIC) enrollment at the last screening encounter among patients who were offered a referral with a documented response (N=438)^a^.

Patient characteristics	Overall (N=438), OR^b^ (95% CI)	Academic clinic (n=384), OR (95% CI)	Nonacademic clinics (n=54)^c^, OR (95% CI)
Sex (Ref: female)			
Male	1.04 (0.67-1.64)	0.99 (0.61-1.61)	7.28 (0.55-95.53)
Race and ethnicity (Ref: non-Hispanic White)			
Non–Hispanic Black	1.03 (0.44-2.42)	0.85 (0.31-2.30)	Ref
Hispanic	1.68 (0.71-3.90)	1.28 (0.49-3.37)	1.99 (0.77-5.15)
Newborn (Ref: no)			
Yes	6.80 (3.89-11.86)^d^	7.79 (4.46-13.63)^d^	1.16 (0.20-6.67)
Preferred language (Ref: English)			
Spanish	1.22 (0.67-2.22)	1.31 (0.71-2.46)	0.41 (0.07-2.48)
Insurance (Ref: non-Medicaid)			
Medicaid	1.33 (0.46-3.89)	2.06 (0.71-5.92)	0.06 (0.01-0.72)^d^
Year at the first screening (Ref: 2020‐2021)			
2022‐2023	1.17 (0.70-1.97)	1.42 (0.83-2.41)	0.08 (0.00-1.60)
Academic clinic (Ref: no)			
Yes	13.29 (4.77-37.02)^d^	—^e^	—

a Multivariable logistic regression adjusted for child age, sex, race and ethnicity, preferred language, insurance type, year of first screening, clinic type, and days between first and last visits; it was not statistically significant and is therefore not shown. Other race and ethnicity and other language categories are suppressed due to small sample sizes.

b OR: odds ratio.

c In the nonacademic clinic model, the non-Hispanic White category was omitted due to zero observed WIC enrollments. The analytic sample size remained unchanged (n=54).

d These estimates indicate statistical significance at *P*<.05.

e Not applicable.

## Discussion

### Principal Findings

Our findings indicate that patient characteristics associated with WIC participation vary across stages of engagement in the WIC referral continuum, including initial enrollment status, referral acceptance, and subsequent enrollment. Consistent with prior literature, we found that non-Hispanic White race, non-Medicaid insurance, and attending a nonacademic clinic were associated with a higher likelihood of not being enrolled in WIC at the first screening encounter. We also observed higher odds of nonenrollment in later years of the study period, which may reflect changes in screening implementation across clinics or broader fluctuations in WIC participation during the COVID-19 pandemic recovery period. Among patients who were not enrolled at the first screening, most accepted a referral when offered, with higher odds of referral acceptance observed among non-Hispanic Black and Hispanic patients, newborn-aged patients, and those with Medicaid insurance. In contrast to patterns observed for nonenrollment, patients seen in academic clinics were more likely to accept a referral and to subsequently enroll in WIC.

These differences may reflect variation in screening workflows and counseling context, as providers in the academic clinic conducted screening directly within the visit note template and may have had more opportunities to discuss WIC benefits with caregivers. Among those who accepted a referral and had a documented follow-up response, newborn-aged patients and those receiving care in academic clinics had substantially higher odds of enrollment. Although subgroup estimates should be interpreted cautiously due to sparse data, in nonacademic clinics, Medicaid insurance was associated with lower odds of subsequent enrollment compared with non-Medicaid coverage. Sex and screening year were not significantly associated with referral acceptance in the overall model. Preferred language showed a modest association with acceptance, although estimates varied across clinic-specific models. Together, these findings highlight important differences in engagement across the WIC participation continuum and underscore the influence of clinical context and patient characteristics on both referral uptake and successful enrollment. Conceptualizing these steps as a continuum highlights potential points where health systems may intervene to improve participation, from identifying unenrolled families to supporting successful referral follow-through.

For practices planning implementation of a WIC screening and referral tool, screening modality and workflow integration are key considerations. In our sample, slightly more than a third of eligible participants in the nonacademic clinics completed WIC screening (1108/5385, 38%), suggesting meaningful implementation barriers. Although we attempted to standardize workflows through on-site education and partnership with clinic managers, some clinic staff may have viewed screening for FNP participation as outside their scope of practice or as potentially stigmatizing as demonstrated in our prior work, which could have contributed to higher rates of incomplete screening [24]. Additionally, nonacademic clinics relied on an automated EHR alert and screening during rooming—typically by MAs—rather than embedding screening within the provider note template, which could have contributed to differences in uptake of the screener. Prior studies have demonstrated alert fatigue and high override rates in clinical decision support systems [27-29], and our prior qualitative research similarly suggests challenges with alert timing and navigating workflows [24]. Future research could explore alternative methods for effective screening and workflow using an implementation science framework.

Screening during the clinical encounter by the health care provider may also provide the opportunity for more effective patient education about the benefits of WIC, given the knowledge gap among eligible WIC participants [30]. Caregivers report a high level of trust in their pediatricians, and their decision to enroll in the FNP can be influenced by a discussion with their pediatrician [31]. One prior study demonstrated that integrating WIC services into pediatric well-child and obstetric visits yielded 748 new WIC enrollments over a 21-month period, and the program was positively received by participants, staff, and providers [23]. More research is needed on integrating WIC referrals into clinical settings. These considerations may be particularly relevant given that referral acceptance and subsequent enrollment were higher in the academic clinic setting in adjusted models, underscoring the potential importance of workflow and counseling context.

Although consistent with national and state data, our findings suggest that determinants of WIC engagement differ across stages—from baseline participation to referral uptake and subsequent enrollment—highlighting opportunities for targeted strategies [6]. In particular, eligible non-Hispanic White families were least likely to report WIC participation at the first screening and less likely to accept a referral when offered, suggesting a need for tailored outreach and counseling to address barriers such as stigma or perceived lack of need. Prior research has elucidated potential reasons for WIC nonparticipation among this population, including stigma, feeling as if the program benefits are not needed, or worrying about taking away benefits from others [8,22,32]. Future studies should examine how participation in SNAP, including SNAP-only enrollment and changes in SNAP benefits over time, may influence acceptance of WIC referrals and subsequent enrollment decisions.

### Policy Implications

There are several policy implications for this research. As health care systems increasingly adopt screening and interventions for health-related social needs, policies are needed that incentivize health care systems and clinicians to communicate with federal assistance programs such as WIC. Building EHR tools for WIC screening and referrals may not be feasible for all health care systems; policies are needed that incentivize EHR vendors to build these tools centrally, so they easily integrate with community partners to specifically address pediatric needs [33]. Furthermore, given that the data systems for patients and WIC clients are separate and unable to communicate with one another, data sharing policies could be modified to reduce the administrative burden of communication [34]. To support these policies, future research is needed to determine whether (1) EHR-based screening and automated referral improve enrollment and retention in WIC and (2) WIC referral by health care systems improves health outcomes. Our next steps for this study include optimizing the screening and referral tool workflow to expand across the entire health care enterprise, including additional counties and pediatric, family medicine, obstetrics, and gynecology clinics.

### Limitations

There are several limitations to this research. First, these findings describe a specific EHR-based intervention implemented in 8 clinics in one geographic area. Results may not be generalizable to other settings. Second, FNP participation data were self-reported, and given the inability to verify enrollment in WIC for all participants, it is possible that errors existed in the self-reported data. However, preliminary unpublished data included a validation sample, indicating high accuracy between participants’ responses and actual WIC participation, as verified by our county’s WIC program. Furthermore, SNAP participation was not included as a covariate because the outcome definition of WIC enrollment included participants receiving both WIC and SNAP, creating overlap between the covariate and the outcome variable. Third, given the way the EHR screening tool was implemented in the academic clinic using the progress note template, if the responses were not documented, we could not calculate the percentage of patients who were not screened, which may introduce selection bias. Finally, we can only comment on associations between patient demographic characteristics collected in the EHR and FNP participation screening responses, WIC referral acceptance, and WIC enrollment. These associations are not necessarily causal, but we hope these data will inform future work as we continue to grow these datasets and optimize clinical workflows in our health care system.

### Conclusions

A primary care, EHR-based WIC screening and referral tool can facilitate screening and referral to WIC. Direct, automated referrals to WIC from health care settings are an innovative method for potentially improving access to WIC and promoting health equity. Future research should examine the effectiveness of health care–based referrals on WIC enrollment and retention, the most effective method of referral from health care systems, and the intervention’s impact on health outcomes. Qualitative research could explore clinical and patient perceptions to ensure equity in exploring differences by race and ethnicity.

## Abbreviations

EHR: electronic health record
FNP: Federal Nutrition Program
MA: medical assistant
OR: odds ratio
SNAP: Supplemental Nutrition Assistance Program
WIC: Special Supplemental Nutrition Program for Women, Infants, and Children

## References

[1] Venkataramani M, Ogunwole SM, Caulfield LE (2022). Maternal, infant, and child health outcomes associated with the Special Supplemental Nutrition Program for Women, Infants, and Children. Ann Intern Med.

[2] Daepp MIG, Gortmaker SL, Wang YC, Long MW, Kenney EL (2019). WIC food package changes: trends in childhood obesity prevalence. Pediatrics.

[3] Burke MP, Jones SJ, Frongillo EA, Fram MS, Blake CE, Freedman DA (2018). Severity of household food insecurity and lifetime racial discrimination among African-American households in South Carolina. Ethn Health.

[4] Shaker Y, Grineski SE, Collins TW, Flores AB (2023). Redlining, racism and food access in US urban cores. Agric Human Values.

[5] Kessler C, Bryant A, Munkacsy K (2025). National- and State-Level Estimates of WIC Eligibility and Program Reach in 2023. https://www.fna.usda.gov/sites/default/files/resource-files/wic-eer2023-report.pdf.

[6] Kessler C, Bryant A, Munkacsy K, Gray KF (2024). National and State Level Estimates of WIC Eligibility and Program Reach in 2022. https://www.fna.usda.gov/research/wic/eer/2022.

[7] Davis RA, Leavitt HB, Chau M (2022). A review of interventions to increase WIC enrollment and participation. J Community Health.

[8] Gago CM, Wynne JO, Moore MJ (2022). Caregiver perspectives on underutilization of WIC: a qualitative study. Pediatrics.

[9] Henchy G (2019). Making WIC work better: strategies to reach more women and children and strengthen benefits use. https://frac.org/wp-content/uploads/Making-WIC-Work-Better-Full-Report.pdf.

[10] Jacobs K, Adeniran O (2022). WIC during COVID-19: participation and benefit redemption since the onset of the pandemic. https://frac.org/wp-content/uploads/wic-during-covid-19-2022.pdf.

[11] Agyapong E, Vasan A, Anyigbo C (2024). Reducing WIC administrative burdens to promote health equity. JAMA Pediatr.

[12] Kreider B, Pepper JV, Roy M (2016). Identifying the effects of WIC on food insecurity among infants and children. South Econ J.

[13] Metallinos-Katsaras E, Gorman KS, Wilde P, Kallio J (2011). A longitudinal study of WIC participation on household food insecurity. Matern Child Health J.

[14] Cho SJ (2022). The effect of aging out of women, infants, and children on food insecurity. Health Econ.

[15] Gitterman BA, Chilton LA, Cotton WH (2015). Promoting food security for all children. Pediatrics.

[16] (2021). Linking WIC for health equity: expanding access to WIC through horizontal integration. https://calwic.org/wp-content/uploads/2021/07/Expanding-Access-to-WIC-Through-Horizontal-Integration_07_21.pdf.

[17] (2022). WIC guide for health care providers. https://frac.org/wp-content/uploads/FRAC_AAP_WIC_2.pdf.

[18] Kling SM, Harris HA, Marini M (2020). Advanced health information technologies to engage parents, clinicians, and community nutritionists in coordinating responsive parenting care: descriptive case series of the Women, Infants, and Children Enhancements to Early Healthy Lifestyles for Baby (WEE Baby) Care randomized controlled trial. JMIR Pediatr Parent.

[19] Savage JS, Moore AM, Kling SMR (2023). Coordination between primary care and women, infants, and children to prevent obesity for infants from low-income families: a pragmatic randomized clinical trial. Child Obes.

[20] Bailey-Davis L, Kling SMR, Cochran WJ (2018). Integrating and coordinating care between the Women, Infants, and Children Program and pediatricians to improve patient-centered preventive care for healthy growth. Transl Behav Med.

[21] Budge M, Sharifi M, Maciejewski KR (2023). A mixed-methods analysis of a Special Supplemental Nutrition Program for Women, Infants, and Children and primary care partnership to promote responsive feeding for infants in group well-child care. Acad Pediatr.

[22] Monroe BS, Rengifo LM, Wingler MR (2023). Assessing and improving WIC enrollment in the primary care setting: a quality initiative. Pediatrics.

[23] Goldstein B, Steiner A, VanderWielen L, Bennett K, Tomcho M (2024). Integration of Special Supplemental Nutrition Program for Women, Infants, and Children (WIC) in primary care settings. J Community Health.

[24] McCall A, Strahley AE, Martin-Fernandez KW (2024). WIC staff and healthcare professional perceptions of an EHR intervention to facilitate referrals to and improve communication and coordination with WIC: a qualitative study. J Clin Transl Sci.

[25] Anderson CE, Martinez CE, Ritchie LD (2022). Longer Special Supplemental Nutrition Program for Women, Infants, and Children (WIC) participation duration is associated with higher diet quality at age 5 years. J Nutr.

[26] Seth JG, Isbell MG, Atwood RD, Ray TC (2015). Addressing language barriers in client-centered health promotion: lessons learned and promising practices from Texas WIC. Health Promot Pract.

[27] Ancker JS, Edwards A, Nosal S (2017). Effects of workload, work complexity, and repeated alerts on alert fatigue in a clinical decision support system. BMC Med Inform Decis Mak.

[28] van der Sijs H, Aarts J, Vulto A, Berg M (2006). Overriding of drug safety alerts in computerized physician order entry. J Am Med Inform Assoc.

[29] Van Dort BA, Zheng WY, Sundar V, Baysari MT (2021). Optimizing clinical decision support alerts in electronic medical records: a systematic review of reported strategies adopted by hospitals. J Am Med Inform Assoc.

[30] Terry AL, Wambogo E, Scanlon KS (2024). Reasons for nonparticipation in the Special Supplemental Nutrition Program for Women, Infants, and Children. JAMA Pediatr.

[31] (2023). Pediatricians are essential supports for families: stanford center on early childhood. https://rapidsurveyproject.com/wp-content/uploads/2024/11/RAPID-Pediatricians-factsheet-231219.pdf.

[32] Liu CH, Liu H (2016). Concerns and structural barriers associated with WIC participation among WIC-eligible women. Public Health Nurs.

[33] Dufendach KR, Lehmann CU, Spooner SA, Council on Clinical Information Technology (2024). Special requirements of electronic health record systems in pediatrics: clinical report. Pediatrics.

[34] Drewry MB, Yanguela J, Khanna A (2023). A systematic review of electronic community resource referral systems. Am J Prev Med.

